# Dietary patterns associated with body mass index (BMI) and lifestyle in Mexican adolescents

**DOI:** 10.1186/s12889-016-3527-6

**Published:** 2016-08-22

**Authors:** Laura Elisa Gutiérrez-Pliego, Eneida del Socorro Camarillo-Romero, Laura Patricia Montenegro-Morales, José de Jesus Garduño-García

**Affiliations:** 1 Facultad de Medicina Universidad Autónoma del Estado de México, Av. Paseo Tollocan 248 Universidad, Toluca Estado de México, CP50130 Mexico; 2 Centro de Investigación en Ciencias Médicas UAEMex, Cuerpo académico salud del Universitario Av. Jesús Carranza 205, Universidad, Toluca Estado de México, CP 50130 Mexico; 3 Instituto Mexicano del Seguro Social HGR251, Metepec Estado de, Mexico

**Keywords:** Dietary patterns, Adolescence, BMI, Lifestyle habits

## Abstract

**Background:**

The objetive in this study is to determine the relationship between dietary patterns, BMI, type 2 diabetes mellitus family history (T2DMFH) and some lifestyle variables such as smoking and skipping breakfast in a Mexican adolescent population.

**Methods:**

Cross-sectional, observational, analytical study.Subjetcts: 14-16 years old male and female adolescents (n 373). A previously validated food frequency questionnaire (FFQ) was used and dietary patterns were derived using principal component analysis (PCA). Scores for dietary patterns were categorized by tertiles.

**Results:**

Three major dietary patterns that explained 47 % of variance were found: westernized, high in protein/fat and prudent pattern. Subjects at the highest tertile of prudent pattern had lower BMI. And was also associated with less T2DMFH and less smoking habit when compared with the lowest tertile. We found a positive correlation between BMI and high scores for westernized and high in protein/fat pattern

**Conclusions:**

Dietary patterns of adolescents are a public health concern because there is a direct association between inadequate diet at this early age and obesity

## Background

Dietary patterns emerged as an alternative method for the study of the relationship between diet and chronic diseases; traditionally, nutritional epidemiology studies are focused on some diseases and their relation to specifics nutrients or foods [1, 2], moreover people consume food combinations that contain a mixture of nutrients. Dietary patterns analysis focuses on the study of the diet as a whole concept. Its advantage relies in allowing a more complete vision of the diet and its influence on health outcomes, in addition to facilitate comparison between dietary exposures in different cultural groups and population [1–3].

The most common methods used to analyze dietary patterns are: factor analysis (FA), cluster analysis (CA), reduced rank regression (RRR), and principal component analysis (PCA). PCA is the most used trough the world to associate dietary patterns with chronic diseases. Validated food frequency questionnaires (FFQ) and food intake reports are instruments used to derive dietary patterns [2].

Most of the studies regarding dietary patterns have been performed in adult populations. There are few reports in adolescents. It has been suggested that unhealthy dietary patterns are present from early stages of life. The impact on unhealthy outcomes like overweight, obesity or some other metabolic disorder are still controversial in children and adolescents [4–6]. Despite being widely recognized the importance of establishing healthy eating behaviors early in life, few investigations have been made on dietary patterns in adolescents [7]. This study aims to understand the dietary patterns of adolescents and their relation to different lifestyle variables and body mass index (BMI).

## Methods

A cross-sectional, observational, analytic study was conducted in the Medical Sciences Research Center (CICMED, for its acronym in spanish). We invited to participate all first year students, in two high-schools belonging to the Autonomous University of the State of México.

Subjects who accepted to participate were scheduled to an appointment. Parents or guardians were given an informed consent letter to be signed. A physical examination was performed and they were asked to answer a previously validated in Mexican population FFQ [8].

### Anthropometric measurement

Weight was obtained with a Tanita® scale, under fasting conditions, with minimal clothing and without shoes, in an upright position, heels together and toes slightly apart. Height was obtained using a Seca® stadiometer placed on the wall forming a 90° angle with the floor, participant was standing in Frankfort plane, without shoes or any object on the head.

The BMI was calculated using data from weight and height (BMI = weight/heigh^2^) and expressed in kg/m^2^. The cutoffs used for the study were those proposed by Mercedes de Onis for the World Health Organization (WHO) in 2007 [9] which defines overweight as +1 standard deviation (SD) and obesity as +2SD.

### Dietary patterns assessment

A FFQ was given to be answered and the statistical method of principal component analysis (PCA) was used. The questionnaire gathers data of consumption from 116 food items. For each food, a common ration was assigned (e.g. 1 tortilla, 1 apple, etc). Subjects reported the frequency of consumption of such foods in the last six months using ten options of response going from “never” to “6 or more times per day”. Answers were transformed to average daily intake.

For PCA, each questionnaire responses were coded with a value of 0-9 (0 = never, 1 = less than one per month, etc). Food items were categorized into 28 groups based in other studies [10, 11] according to the similarity of nutrients (e.g whole grains, fruits, and desserts) and others were considered individually (e.g tortilla). The answers for each food groups were summed and standardized using Z-score. After that, PCA was carried out using orthogonal transformation (varimax rotation) to reduce the variance, determine dietary patterns and the factor loadings for each food group. Components with an eigenvalue ≥1.5 were retained and factor loadings > .04 were used to describe the component. Finally, dietary patterns were named based on the literature [5, 10–13]. Subsequently, the factor scores for each participant were used to determine the dietary pattern followed by each subject, and also categorized by tertiles for further analysis.

### Other variables

The presence of type 2 diabetes mellitus family history (T2DMFH) was considered if the participant reported that one or more of the parents or grandparents had DM2. Smoking was considered as a minimum consumption of one cigarette per week in the past six months. And skipping breakfast was considered for this study as the lack of breakfast in 4-7 days a week in the past six months.

### Statistical analysis

A descriptive analysis of the studied variables was conducted using means and standard deviations for continuous variables. Qualitative variables were expressed as percentage. One-way ANOVA was used to determine differences between BMI and weight across each dietary patterns’ tertile. For qualitative variables such as T2DMFH, smoking and skipping breakfast, a Chi-square test was used. Pearson and Spearman test was conducted as appropriate for analyzing the correlation between BMI and dietary patterns scores. All tests were statistically significant at *p* < 0.05 and the statistical package used was SPSS 15.0.1.windows Chicago IL USA

## Results

We include 373 participants (160 men, 213 women). The mean age was 14.5 [14–16] years. The mean BMI was 24.1 ± 3.4 kg/m^2^, and a mean calorie intake of 8405.4 ± 2937 kJ/day. According BMI 67 % (250 subjects) had normal weight, 24.3 % (91 subjects) had overweight and 8.5 % (32 subjects) had some obesity level. About 11.8 % (44 subjects) reported smoking; 65 % (242 subjects) reported frequent fasting and 37.5 % had at least one close relative (parents or grandparent) with DM2 (Table 1)

**Table 1:** Baseline characteristics

	n	%
n	373	
Male	160	42.9
Female	213	57.1
Normal weight	250	67.0
Overweight	91	24.3
Obesity	32	8.5
Smoking	44	11.8
Skipping breakfast	242	64.9
2DM Family history	140	37.5
	Mean	SD
Age, *years*	14.5	0.61
Weight, *kg*	62.2	10.1
Height, *cm*	160	5
BMI, *kg/m* ^*2*^	24.1	3.4
Intake^a^, *kJ/day*	8405.4	2937

*kJ* = kilojoules (1 kcal = 4.184 kJ)

^a^Daily intake was calculated according to the FFQ

.

To analyze the validity of the data from each questionnaire, the extreme studentized deviation (ESD) [14] was applied and those questionnaires whose caloric intake was between 2,092 and 27,196 kJ/day were taken as valid.

Prior to PCA, a Kaiser-Meyer-Olkin test of sampling adequacy (.888) and a Bartlett test of sphericity (*p* < 0.01) was performed to assess whether the factor model as a whole was significant.

Three components or dietary patterns were obtained and named as: 1) Westernized pattern: High intake of refined cereals, snacks, desserts, sweets and sugar, pastries, soda. 2) High in protein/fat pattern (HPF): High consumption of eggs, poultry, red meats, sausages and alcohol. 3) Prudent pattern: High consumption of vegetables, legumes, nuts and seeds, fruits and whole grains. These three major patterns explained 47.3 % of the total variance (Table 2).

**Table 2:** Factor loadings of dietary patterns in Mexican adolescents^a^

Food groups	Component or pattern		
	Westernized	High animal protein and fat	Prudent
Refined grains	.755		
Desserts	.685		
Sweets and sugar	.682		
Pastry	.681		
Snacks	.664		
Tortilla	.629		
Mexican food	.570	.444	
High-fat dairy	.532		
Soda	.488		
Poultry	.485	.409	
Egg	.478		
Coffee and tea			
Red meat		.637	
Fish and sea food		.634	
Alcohol		.548	
Low energy beverages		.536	
Processed meat	.473	.523	
Sweet beverages	.415	.503	
Saturated fat		.494	
Whole grains			.610
Low-fat dairy			
Vegetables			.843
Tomato			.703
Potato			.694
Legumes			.692
Fruit			.679
Vegetable oils			.644
Juice		.431	.481

^a^Extraction method: Principal component analysis (PCA) with varimax rotation

Participants in the highest tertiles of Westernized pattern and HPF pattern showed to have a higher BMI compared with those in the highest tertile of the prudent pattern.

As for smoking habit, there was no difference between 1st and 3rd tertiles in unhealthy patterns (westernized and HPF), however, there was less prevalence of smokers in 3rd tertile than 1st tertile of prudent pattern.

It was also found that those participants located in the highest tertile for prudent pattern skipped breakfast more frequently than those at the highest tertiles of unhealthy patterns.

The presence of T2DMFH is associated with unhealthy patterns, it is more common in highest tertiles of unhealthy compared to the highest tertile of prudent pattern (Table 3).

**Table 3:** Characteristics of sample across tertiles of dietary patterns

	Westernized			High animal protein and fat			Prudent		
	T1	T3	*P* value	T1	T3	*P* value	T1	T3	*P* value
Female %	58.00	52.40	0.39	60.40	54.00	0.58	58.80	60.40	0.63
Smoking %	12.00	12.00	0.90	11.20	14.50	0.40	20.90	4.00	0.00
Frequent fasting %	74.10	58.00	0.02	75.00	60.40	0.02	66.90	71.70	0.03
DM2 FH %	32.20	41.10	0.31	33.80	41.10	0.40	46.70	33.80	0.03
OW/Ob %	24.10	46.70	0.00	25.80	46.70	0.00	59.60	9.60	0.00
Weight, kg	59.3±9.1	66.9±10.1	0.00	60.4±9.4	66.2±10.7	0.00	68.2±10.5	56.1±8.3	0.00
BMI, kg/m2	23.1±3.0	26.2±3.9	0.00	23.4±3.1	25.5±3.8	0.00	26.4±3.6	21.8±3.4	0.00

*T1*: Tertile 1

*T3*: Tertile 3

*OW/Ob*: Overweight/Obesity

Chi-square test for qualitative data and one way-ANOVA for quantitative data

Finally, the Pearson correlation analysis between BMI and the different dietary patterns scores showed a positive correlation with the westernized pattern (*r* = .316 *p* < 0.01) and the HPF pattern (*r* = .307 *p* < 0.01). In contrast, a negative correlation was found for the prudent dietary pattern (*r* = -.576 *p* < 0.01) (Table 4).

**Table 4:** Pearson correlation between BMI and dietary patterns scores

Pearson correlation		BMI
Westernized score	R	0.316^a^
	p	<0.01
	N	373
HAPF score	R	0.307^a^
	p	<0.01
	N	373
Prudent score	R	-0.576^a^
	p	<0.01
	N	373

^a^Correlation is significative at 0.01 (bilateral)

## Discussion

In the present study we found that there is a strong relationship between unhealthy dietary patterns (westernized and HPF) and higher BMI in adolescents. In the same way this unhealthy patterns are associated with inadequate eating and lifestyle habits. It was also found that a large number of participants with T2DMFH were following an unhealthy dietary pattern.

Obesity and overweight are considered a global epidemic. As the prevalence of these diseases in children and adolescents is increasing every day in both developed and developing countries [15]. Obesity represents a critical risk factor for many chronic diseases such as type 2 diabetes, cardiovascular disease, hypertension, dyslipidemia, and some type of cancers. Obesity by itself, could be consider a risk factor for mortality. There is an straight correlation between the severity of overweight and mortality risk [16]. From the public health view obesity represents one of the more important or maybe the most important challenge. The International Obesity Task Force estimates the costs of obesity between 2 and 8 % of total health budget [17] In Mexico, the total cost of overweight and obesity (direct and indirect cost) in 2008 was of 67.345 million Mexican pesos and is expected to rise by 2017 to 150.860 million Mexican pesos [18]. Data from the National Survey of Health and Nutrition 2012 (ENSAUT for its acronym in Spanish) reveal that in Mexico, the prevalence of overweight and obesity in adolescents between 12 and 19 years old was 35 %, i.e. 1 in 5 adolescents are overweight, and 1 in 10 are obese [19]. When analyzing the prevalence of overweight and obesity in this study, a similar data was found (32.9 %), which places this population slightly below the national mean. It is extremely relevant to describe all phenomena related to obesity in Mexican adolescents. It has been described that Mexican children and adolescents has the higher increasing trend in the prevalence of obesity and overweight all over the world [20]. The most serious consequences of obesity in adolescents will appear until adulthood (out of 3 obese children, one adult will remain so) because of severe comorbidities [21].

Overweight and obesity results from the interaction of different genetic, environmental and lifestyle factors [22] Considering the genetic factor, it is known that if both parents are obese, the risk of obesity in children will be from 69-80 % when only one parent is obese the risk will be of 41-50 % and conversely if neither parent is obese, the risk decreases to 9 % [23]. However it is considered that the most important factor in the development of such diseases corresponds to lifestyle, specially diet and physical activity have important influence on the development of overweight and obesity [24].

One aspect closely linked to obesity is undoubtedly food. Human consumption is an act in which diverse phenomena of nature are combined, as it includes a set of social and biological interactions mediated by the culture in which it takes place [25]. Eating habits in adolescence has a biological and cultural nature, the first one is related to growth and biological development and the second with formation of beliefs and daily practices according to social influences such as friends, school, media and family. Both aspects determine current and future stages of teenage life [26]. During adolescence, many physical and psychological changes occur that modifies dietary patterns and physical activity habits, which means that if a good match between the needs (as a consequence of age, development and new lifestyle) and the energy intake does not occur, teens will have a great likelihood of eating disorders either by default (anorexia, bulimia) or excess (overweight and obesity) [27]. In the present study we can identify certain meal habits that are present since this early stages of life. And we also describe how this habits are associated with weight.

The analysis of dietary patterns emerged as an alternative to the study of the diet and its relation to various health outcomes; however, these studies have been done mostly in adults and mayors, finding consistency in dietary patterns derived. Regarding dietary patterns found in previous studies performed in adult populations in the world. It has been identified three consistent dietary patterns: the prudent or healthy dietary pattern characterized by a high consumption of fruits , vegetables, whole grains and fish [28–42]; the westernized or unhealthy patter characterized by low consumption of fruits, vegetables and high consumption of refined grains, processed food, pastry and high-fat diary [33–35, 37–40, 43–52]; and a third dietary patter called High in protein/fat pattern defined by a high intake of dairy, eggs, red meat, processed meat, poultry and low consumption of fruits, vegetables and whole grains [29, 32, 36, 41, 42, 45, 53–58]. This study is one of the first made in adolescent population, and has identified three major dietary patterns similar to those found in other studies in adults. The similarity found between dietary patterns of adolescents and adults suggests that behaviors early in life remain largely unchanged until adulthood.

By analyzing the relationship between BMI and dietary patterns, it was found that the higher the score of unhealthy patterns, the higher the BMI, and conversely, the higher the score in prudent pattern, the lower was the BMI. This results suggest that although weight gain is determined by food consumption from a quantitative view, it is also the qualitative aspect which needs more attention.

The present study provides evidence that adolescents with higher scores of unhealthy dietary patterns are not only associated with weight, another cardiovascular risk factors like smoke are seen in patients with higher scores of unhealthy patterns. These results are consistent with a study that described that teens who smoked tended to eat fewer vegetables, dairy and exercise less [59]. The results of this study show that a high percentage of the population has the habit of skipping breakfast (regardless of dietary pattern to follow). The literature states that skipping breakfast is a risk factor for being overweight or obese and it is possible that the information that adolescents have about the consequences of skipping breakfast is incomplete or wrong [60, 61].

It is known that the family has a strong influence on the diet of children and adolescents and also on their food-related behaviors. This influence can have a significant impact on weight gain. The association of the presence of T2DMFH and unhealthy patterns can be explained considering that each cultural group transmits their eating habits from generation to generation through food education to their children, regardless of whether is healthy or not [62].

The strength of this study is that is one of the first studies designed to analyze dietary patterns in adolescents and it is performed in one of the most high prevalence of obesity population in the world. The weakness of this study is the cross sectional design.

## Conclusions

Dietary patterns of adolescents are a public health concern because there is a direct association between inadequate diet at this early age and obesity. Therefore, adolescence is a good stage to intervene in changing dietary patterns and the consolidation of a healthy lifestyle that the teenager will keep until adulthood and which in turn will transmit to future generations. With advances in the study of dietary patterns, the development of effective strategies focused on changing patterns that could provide a clear starting point in the prevention and treatment of various diseases such as overweight and obesity in adolescents should emerge.

## Abbreviations

BMI: Body Mass Index
CA: Cluster Analysis
FA: Factor Analysis
FFQ: Food Frequency Questionaries’
HPF: High in Protein/Fat Pattern
PCA: Principal Components Analysis
RRR: Reduced Rank Regression
SD: Standar Deviations
T2DMFH: Type 2 diabetes Family History
